# Acute testosterone administration does not affect muscle anabolism

**DOI:** 10.1186/s12986-019-0385-0

**Published:** 2019-08-22

**Authors:** David D. Church, Stefan M. Pasiakos, Robert R. Wolfe, Arny A. Ferrando

**Affiliations:** 1 Department of Geriatrics, Donald W. Reynolds Institute on Aging, Center for Translational Research in Aging & Longevity, University of Arkansas for Medical Sciences, Little Rock, AR 72205 USA; 2 Military Nutrition Division, U.S. Army Research Institute of Environmental Medicine, Natick, MA USA

**Keywords:** Testosterone, Amino acids kinetics, Protein metabolism, Stable isotope tracers, Protein synthesis, Protein breakdown

## Abstract

We previously demonstrated that improved net muscle protein balance, via enhanced protein synthetic efficiency, occurs 5 days after testosterone (T) administration. Whether the effects of T on muscle protein kinetics occur immediately upon exposure is not known. We investigated the effects of acute T exposure on leg muscle protein kinetics and selected amino acid (AA) transport using the arteriovenous balance model, and direct calculations of mixed-muscle protein fractional synthesis (FSR) and breakdown (FBR) rates. Four healthy men were studied over a 5 h period with and without T (infusion rate, 0.25 mg·min^− 1^). Muscle protein FSR, FBR, and net protein balance (direct measures and model derived) were not affected by T, despite a significant increases in arterial (*p* = 0.009) and venous (*p* = 0.064) free T area under the curve during T infusion. T infusion had minimal effects on AA transport kinetics, affecting only the outward transport and total intracellular rate of appearance of leucine. These data indicate that exposing skeletal muscle to T does not confer immediate effects on AA kinetics or muscle anabolism. There remains an uncertainty as to the earliest discernable effects of T on skeletal muscle protein kinetics after initial administration.

## Introduction

The effects of exogenous testosterone (T) administration on muscle protein anabolism and lean body mass accrual are well established. The muscle protein kinetic mechanisms through which T administration improves anabolism are less appreciated. Fasted net muscle protein balance is improved in healthy males following a 5d treatment with an oral T analogue [1] or T injection [2]. Muscle protein synthesis (PS) improves with T in fasted muscle of healthy males [1, 2], in part by improving *synthetic efficiency*, where synthetic efficiency refers to the rate of PS relative to the availablilty of amino acid (AA) precursors. In the post-absorptive state, the essential AA precursors for PS at the whole body level are derived entirely from protein breakdown (PB). In certain tissues and organs, the precursors for PS can be derived from uptake of circulating AA. Improved synthetic efficiency of muscle protein in the post-absorptive state in response to T refers to an increase in the rate of PS relative to the rates of PB and inward AA transport [1, 2]. Greater anabolism is achieved when hyperaminoacidemia accompanies T administration through greater increases in inward AA transport, intracellular AA appearance, and subsequently PS [3]. Enhanced muscle protein synthetic efficiency has also been observed in a severely injured clinical population, as administering T for 2 weeks to severely burned patients improves the synthesis/breakdown ratio. However, unlike healthy adults, synthesis and breakdown are both dramatically upregulated in burn patients [4], thus the increase in protein synthetic efficiency secondary to T administration is due to a maintenance of the rate of PS and a reduction in the rate of PB [5]. As such, our work utilizing T for 5 days or longer demonstrates effects on muscle protein kinetics. Whether the effects of exogenous T on muscle protein kinetics occur acutely upon exposure is not known.

We sought to discern the effects of acute T administration on muscle protein kinetics. The investigation centered on the concept of a potential hormonal-induced change in protein kinetics. For example, muscle anabolism and inward AA transport were upregulated with acute insulin infusion [6]. Whether an analogous response was present with T is not known. Although the primary mechanism of T in skeletal muscle is genomic via the androgen receptor, Estrada and colleagues [7] demonstrated T can stimulate extracellular signal-related kinase 1 and 2, which are involved in cellular growth, within a minute in cultured myotubes. Furthermore, the G-coupled protein receptor GPRC6A, a widely expressed calcium and amino acid sensor, has been implicated in the non-genomic action of T [8]. This question may be relevant to populations who are not generally considered for clinical T treatment and are routinely exposed to acute catabolic stress. More specifically, T may be a viable option to conserve muscle mass and ultimately function in healthy poplutations exposed to extreme stress, such as military personnel, including light infantry and special operations forces, who can experience high energy expenditures, severe energy deficits, sleep deprivation, and environmental stress during trainings and combat operations. These exposures typically last ~ 3–60 days and elicit a marked hypogonadal state and catabolism of lean mass [9–11]. In this context, delineating the acute effects of T on skeletal muscle will help refine future efforts to minimize muscle loss in military personnel [12]. Therefore, the purpose of this study, which was conducted in 1995, was to detail the results of a 5 h (hr) T infusion on muscle protein turnover and AA transport in young males using stable isotope methodology and cross-limb modelling kinetics. We hypothesized that exposure to 5 h of T would confer anabolic effects on skeletal muscle. It is important to note that while we previously referred to the results of this study in a brief review [13], the data were never published. Therefore, the effects of acute exposure to T on muscle protein turnover are undetermined.

## Methods

### Subjects

Four healthy males (28.0 ± 3.6 [SD] yr.; 71.2 ± 4.5 kg; 172.9 ± 8.2 cm) participated in this study. Written consent was obtained on all subjects, and the protocol was approved by the Institutional Review Board at the University of Texas Medical Branch at Galveston (UTMB).

### Infusion protocol

**Fig. 1:**
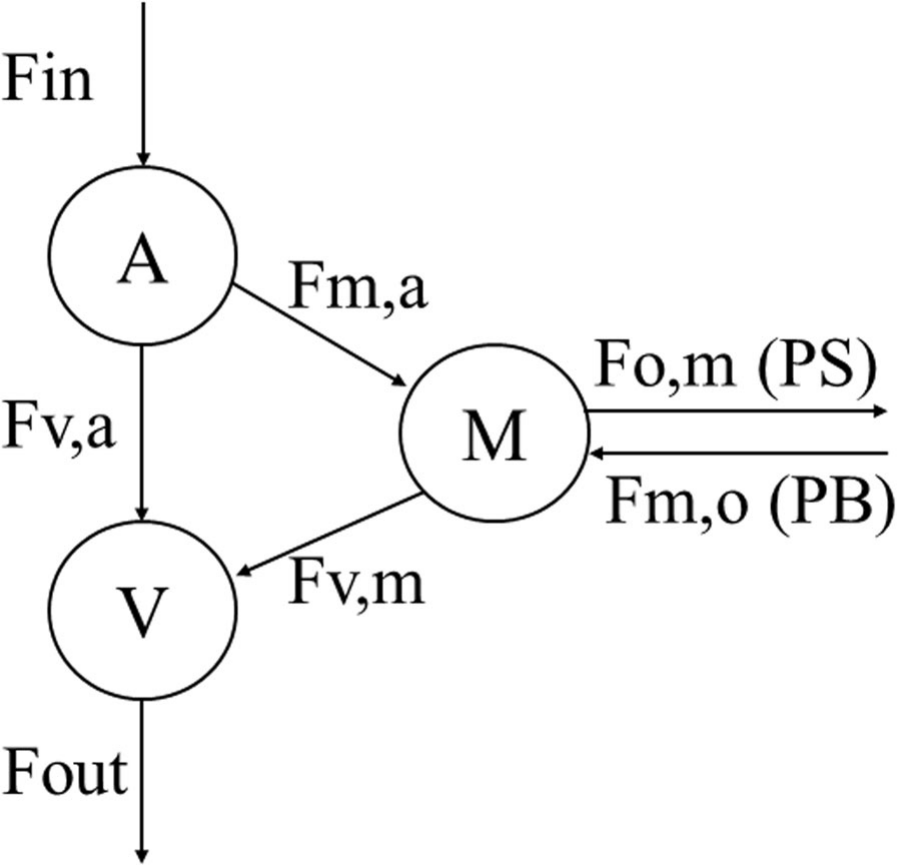
Three-compartmental model of leg amino acid (AA) kinetics. Free AA pools in the femoral artery (A), femoral vein (V), and muscle (M) are connected by arrows indicating unidirectional flow between each compartment. Fin, AA inflow into leg from systemic circulation via femoral artery; Fout, AA outflow from leg via femoral vein; Fv,a, direct AA outflow from artery to vein without entering intracellular fluid; Fm,a, inward AA transport from femoral artery into free muscle AA pool; Fv,m, outward AA transport from intracellular pool into femoral vein; Fm,o, intracellular AA appearance from endogenous sources; Fo,m, intracellular AA utilization

Subjects reported to the Clinical Research Center at the UTMB, Galveston, TX after an overnight fast. Procedures for the cross-limb balance model, and derived kinetic parameters (see Fig. 1), have been outlined in detail previously [2, 14]. Briefly, a 3-Fr 8-cm polyethylene catheter (Cook, Bloomington, IN) was inserted into the femoral vein and another into the femoral artery under local anesthesia. Both femoral catheters were used for blood sampling; however, the femoral arterial catheter was also used for indocyanine green infusion for the determination of leg blood flow. A 20-gauge polyethylene catheter (Insyte-W, Benton-Dickinson, Sandy, UT) was placed in an antecubital vein for infusion of labelled AAs. A second 20-gauge polyethylene catheter was placed in the contralateral wrist and surrounded by a heating pad maintained at ~ 65 °C for measurement of systemic concentration of indocyanine green. Sample analysis and gas chromatography-mass spectrometry (GC-MS) of blood and muscle isotope enrichment was also previously described [15]. Subjects were studied in a cross-over fashion with treatment order (T, intralipid [IL]) randomized.

**Fig. 2:**
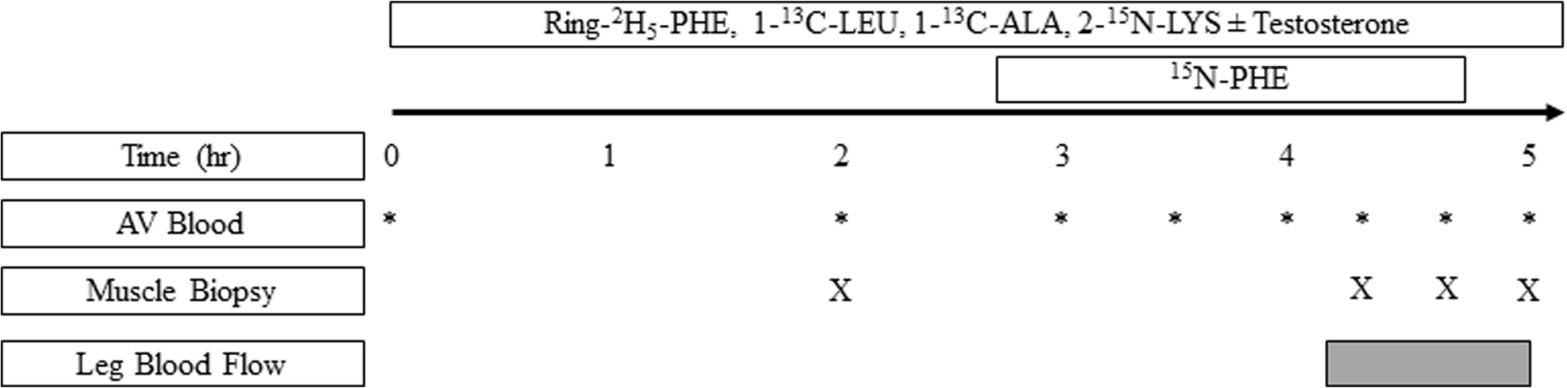
Isotope infusion protocol with or without (±) Testosterone. Ring-^2^H_5_-PHE, L-[*ring*-^2^H_5_] phenylalanine; 1-^13^C-LEU, L-[1-^13^C] leucine; 1-^13^C-ALA, L-[1-^13^C] alanine; 2-^15^ N-LYS, L-[2-^15^ N] lysine; ^15^N-PHE, L-[^15^N] phenylalanine

The infusion study to determine protein kinetics is outlined in Fig. 2. Participants received both an infusion of T and IL, separated by at least five days. For the T infusion, T (Schein Pharmaceutical, Florham Park, NJ) was dissolved into IL (Baxter Healthcare, Deerfield, IL) and infused at a rate of 0.25 mg·min^− 1^; providing 30 mg of T over the 5 h study period. IL alone was infused at the same rate. Considering healthy adult men produce 3.8–9.1 mg T/day [16], the T infusion was devised to expose tissue to supra-physiological amount of bioactive free T concentrations.

Baseline blood samples were obtained for the measurement of background AA enrichment, indocyanine green concentration, and free T concentration. Stable isotopes were concomitantly infused at the following primed (PD) continuous infusion rates (IR) throughout the 5-h study: L-[ring-^2^H_5_] phenylalanine, IR = 0.05 μmol·kg^− 1^·min^− 1^, PD = 2 μmol/kg; L-[2-^15^ N] lysine, IR = 0.08 μmol·kg^− 1^·min^− 1^, PD = 7.2 μmol/kg; L-[1-^13^C] leucine, IR = 0.08 μmol·kg^1^·min-1, PD = 4.8 μmol/kg; L-[1-^13^C] alanine, IR = 0.35 μmol·kg^− 1^·min^− 1^, PD = 35 μmol/kg. After 2 h of infusion (Fig. 2), a PD (2 μmol/kg) continuous (0.05 μmol·kg^− 1^· min^− 1^) infusion of L-[^15^N] phenylalanine was initiated and maintained until the 4th h. The arterial and intracellular L-[^15^N] phenylalanine enrichments at plateau and during the decay were utilized to determine fractional breakdown rate (FBR) [17]. Biopsies of the vastus lateralis were performed at 2 h, 4 h 30 min, 4 h 45 min, and 5 h of infusion. Fractional synthetic rate (FSR) of skeletal muscle protein was determined by the incorporation of L-[*ring*-^2^H_5_] phenylalanine into protein from 2 to 4 h 45 min and also from 2 to 5 h (values averaged). The biopsies at 4 h 45 min and 5 h were utilized to measured intracellular ^15^N-phenylanine enrichment for the determination of FBR.

### Free testosterone concentration

Free testosterone concentrations in serum were determined by a double antibody method with commercial radio immunoassays (Diagnostic Products, Los Angeles, CA), which were standard at that time. The intra-assay CV was 2.9%. The area under the curve (AUC) throughout the entire infusion protocol (0 to 5 h) was calculated using the trapezoidal method.

### Statistical analysis

Data are presented as means ± SEM. All variables were compared by paired samples *t*-test with statistical significance designated at α ≤ 0.05.

## Results

**Fig. 3:**
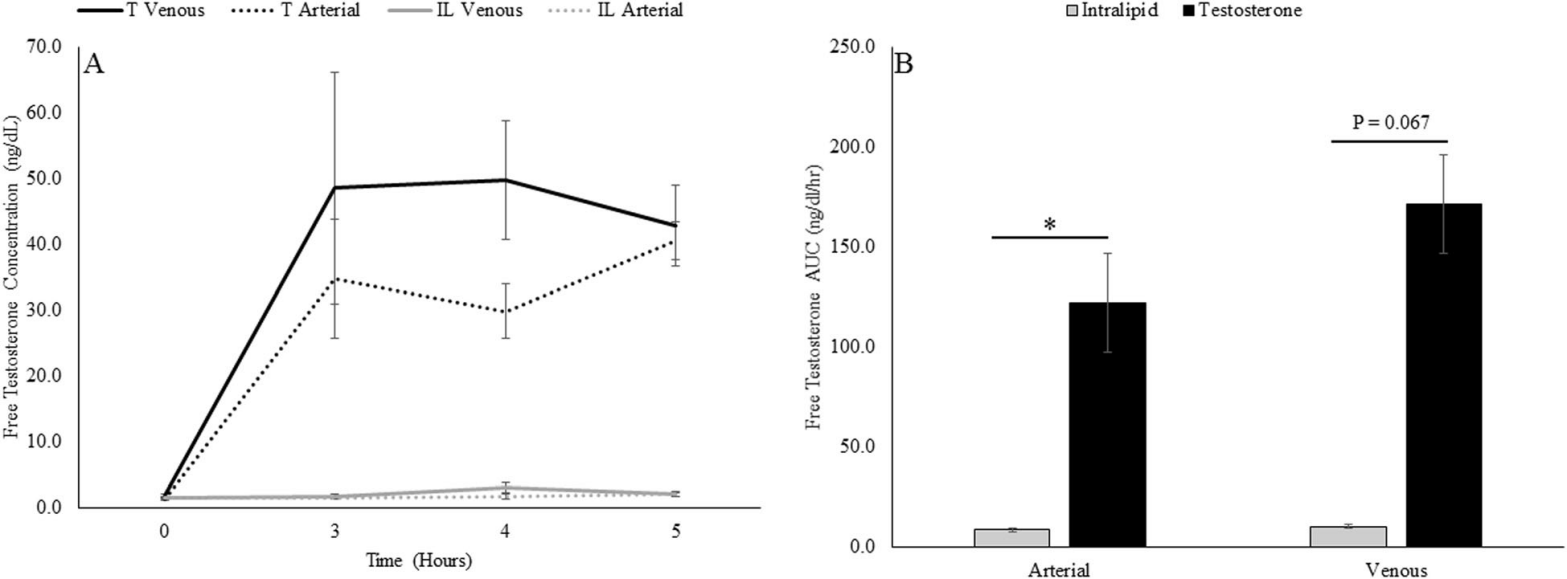
Values are means ± SEM. Temporal (**a**) and Area under the curve (**b**) of arterial and venous free testosterone concentrations during testosterone (black line) and intralipid (grey line) infusion

AA kinetics are presented in Table 1. The temporal arterial and venous free T responses to T and IL infusion can be seen in Fig. 3. During the T infusion protocol the arterial free T AUC (121.7 ± 19.4 ng/dl/hr) was significantly (*p* = 0.009) greater than IL (8.4 ± 1.3 ng/dl/hr), whereas the venous free T AUC (T = 171.3 ± 57.7 ng/dl/hr.; IL = 10.5 ± 2.2 ng/dl/hr) was not different (*p* = 0.064) between trials. As a comparison, the clinical reference range for free T concentrations is 5–9 ng/dl in young men [18]. Thus, tissue was exposed to approximately 12 times the normal biologically active form of T.

**Table 1:** Leg muscle amino acid kinetics

Factor	Phe		LEU		LYS		ALA	
	T	IL	T	IL	T	IL	T	IL
Fin	188 ± 39	194 ± 29	400 ± 86	413 ± 60	855 ± 241	760 ± 118	657 ± 102	686 ± 71
Fout	205 ± 47	210 ± 29	445 ± 112	449 ± 65	932 ± 299	799 ± 131	807 ± 160	786 ± 62
Fv,a	76 ± 38	57 ± 22	195 ± 79	32 ± 21	552 ± 208	384 ± 120	273 ± 45	342 ± 65
Fm,a	113 ± 38	137 ± 32	205 ± 19	381 ± 69	302 ± 43	376 ± 102	384 ± 64	345 ± 20
Fv,m	130 ± 41	153 ± 34	**250 ± 34**	**417 ± 73**	380 ± 104	414 ± 109	533 ± 118	444 ± 11
Fm,o	88 ± 14	99 ± 15	151 ± 37	142 ± 19	295 ± 111	355 ± 61	760 ± 67	715 ± 112
Fo,m	71 ± 10	83 ± 17	106 ± 9	106 ± 10	**217 ± 52**	**317 ± 49**	611 ± 49	616 ± 108
Ram	201 ± 44	236 ± 35	**356 ± 40**	**523 ±** 77	597 ± 150	731 ± 148	1144 ± 125	1060 ± 116
NB	−17 ± 9	−16 ± 3	−46 ± 30	−36 ± 15	−78 ± 63	−38 ± 13	−150 ± 65	−99 ± 22
DNS							2114 ± 186	1988 ± 312

Values are means ± SE and are expressed as nmol · min^− 1^ · 100 ml leg^− 1^*. DNS* de novo synthesis. Bolded values indicate significant differences (*p* ≤ 0.05) between T (testosterone) and IL (intralipid) infusion. Fin, amino acid (AA) inflow into leg from systemic circulation via femoral artery; Fout, AA outflow from leg via femoral vein; Fv,a, direct AA outflow from artery to vein without entering intracellular fluid; Fm,a, inward AA transport from femoral artery into free muscle AA pool; Fv,m, outward AA transport from intracellular pool into femoral vein; Fm,o, intracellular AA appearance from endogenous sources; Fo,m, intracellular AA utilization; Ram = Fm,o + Fm,a, total intracellular AA rate of appearance; NB, net AA balance

**Fig. 4:**
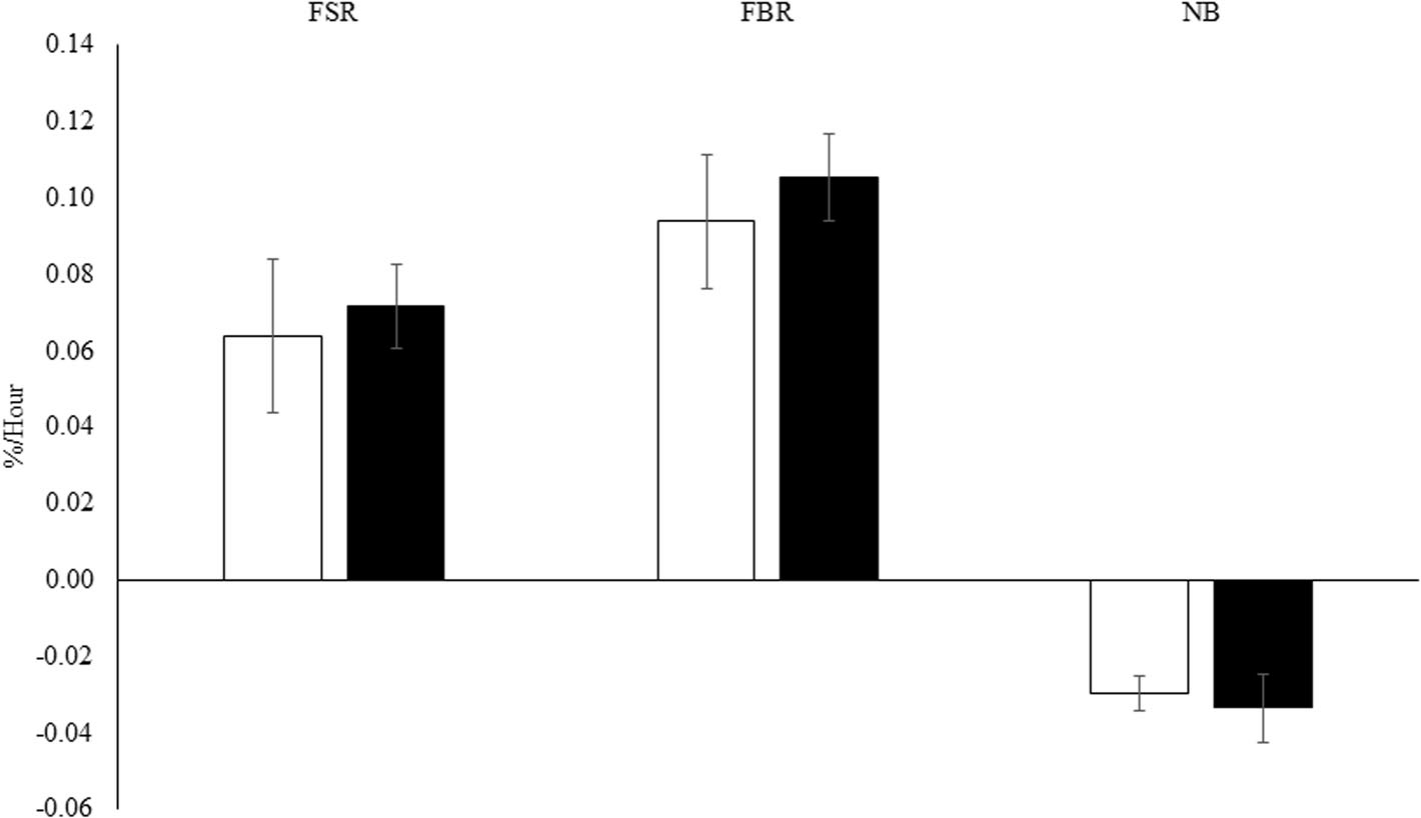
Values are means ± SEM. Fractional synthetic (FSR) and breakdown (FBR) rate as well as net balance (NB) direct incorporation values during testosterone (■) and intralipid (□) infusion

No significant differences (*p* > 0.05) were observed (Fig. 4) for FSR (T = 1.72 ± 0.27; IL = 1.54 ± 0.48%/day), FBR (T = 2.53 ± 0.27; IL = 2.25 ± 0.42%/day), fractional net balance (FSR-FBR; T = − 0.81 ± 0.21; IL = − 0.72 ± 0.12%/day), or leg blood flow (T = 0.23 ± 0.04; IL = 0.23 ± 0.02 L/min). Protein synthetic efficiency (model-derived Fo,m/Ra,m; i.e., synthesis/intracellular AA appearance) was not significantly altered when measured with either Phe (*p* = 0.256; IL = 37.4 ± 9.6%; T = 42.3 ± 7.0%) or Lys (*p* = 0.365; IL = 45.0 ± 3.7%; T = 36.9 ± 4.6%). There were no demonstrated changes in the PS/PB (Fo,m/Fm,o) ratio when measured with Phe (*p* = 0.977; IL = 82.0 ± 5.2%; T = 82.1 ± 6.8%) or Lys (*p* = 0.424; IL = 89.9 ± 2.1%; T = 82.3 ± 9.0%).

There were some demonstrated changes in AA kinetics. The outward transport of leucine from skeletal muscle (*p* = 0.046; IL: 417 ± 37; T: 250 ± 17 nmol · min^− 1^ · 100 ml leg^− 1^) as well as the total intracellular rate of appearance of leucine (*p* = 0.041; IL: 523 ± 39; T: 356 ± 20 nmol · min^− 1^ · 100 ml leg^− 1^) were significantly decreased during T. Intracellular lysine utilization rate was also significantly (*p* = 0.041) increased during IL (317 ± 25 nmol · min^− 1^ · 100 ml leg^− 1^) as compared to T (217 ± 26 nmol · min^− 1^ · 100 ml leg^− 1^). No other significant differences were noted.

## Discussion

These results indicate that, unlike insulin [6], acute tissue exposure to supra-physiological free T does not affect muscle protein and AA kinetics. There were only minor indications of initial action of T that were afforded by the use of several essential AA tracers. The reduction in intracellular leucine appearance, along with a reduction in outward transport from the muscle are consistent with increased muscle oxidation of leucine; however, oxidation was not directly measured. The rationale for the reduction in intracellular AA utilization (PS) of lysine is not clear, as the changes in kinetic parameters or ratios with T were not significantly different. Thus, the novel findings of this study is that only minor alterations in AA kinetics occur during acute T exposure.

Alterations in muscle protein kinetics require multiple events such as changes in translation, inhibition of catabolic signaling, and activation of anabolic signaling pathways to occur [19]. Increased anabolic signaling through mTORC1 via upstream effectors such as IGF-1/Akt and/or ERK1/2 have been hypothesized to contribute to T-mediated increases in protein synthesis [20–22]. A role for the E3 ligases (MuRF1 and MAFbx), TGFβ/myostatin/activin/Smad signaling, and autophagy have all been demonstrated for T-mediated decreases in protein catabolism (Rossetti, 2018). The androgen receptor carries out the genomic actions of T. We observed a significant increase in systemic free T concentrations; however, the absence of T uptake by the muscle most likely prevented the activation of the androgen receptor. This acute exposure of skeletal muscle, as opposed to the prolonged exposure of days, provides a reasonable explanation of our results. It is plausible that the extension of our metabolic measurements to incorporate a longer period after T administration would have demonstrated a net uptake of T, as the free was roughly in balance at the end of the study infusion period. This may also have realized an activation of the androgen receptor’s hypertrophic gene program. The broad action of T and lack of molecular measurements in the present study prevent definitive conclusions about the molecular mechanisms of acute T exposure.

It is interesting to note that the increase in arterial free T was significant, while venous concentrations approached significance. This indicates that skeletal muscle was exposed to supraphysiological free T concentrations. However, the absence of a “net” free T uptake by skeletal muscle may partially explain the absence of anabolic effect. These data may lead to speculation that the chosen study period may have not have been sufficient; however, subject safety concerns regarding this type of administration, now generally thought to be minimal, were given considerable weight at that time. In retrospect, it may have been worthwhile to conduct these kinetic measurements 24 h after T administration to ascertain potential effects.

While the small sample size may seem disconcerting, the study design (paired testing) and proven methodology has the potential sensitivity to discern kinetic effects in studies with small sample sizes [23]. At the time, these results were deemed unremarkable and we subsequently pursued other administration routes (injection, oral) of T. Our results utilizing T injection demonstrated that the fasted ratio of PS/PB improved significantly to virtually 100% after 5 days, indicating that most all the phenylalanine and lysine derived from PB was reincorporated into PS [2]. These data indicate that when T effects manifest, it can effect muscle protein kinetics in the fasted state through an improved synthetic ratio. More specifically, this entails a preferential routing of essential AAs derived from PB to PS.

## Conclusion

The most important aspect highlighted by this research, is that there exists a gap in our experimental knowledge of T effects on muscle protein kinetics. We now know that there are no acute T effects; however, we also know that the earliest demonstrated effects, due to a lack of experimental data, are 5 days after administration [1, 2]. Thus, we have a knowledge gap in terms of the initiation of protein kinetic effects that spans from the time point of administration until 5 days post-administration. This gap has never been of clinical significance, since T administration is normally given for extended periods of time to correct hypogonadal states, or more recently, during hypocaloric states in obese populations [24–26]. Even when utilized in severe burn injury [5], intensive care treatment of this populations entails administration for 1 month or longer [27]. However, the efficacy of T administration in healthy populations exposed to severe catabolic stress for short durations highlights a need to close this knowledge gap. In particular, special operations forces combat training results in a hypogonadal state [11, 28, 29], a loss of lean mass [10, 11, 30], and decreased performance outcomes due to a convergence of many different physiological and environmental stressors [29, 31–34]. Thus, the ability to discern the short-term anabolic potential of T may be of substantial benefit to certain military populations whose occupational demands often include exposure to extreme catabolic stress. The current study indicates that within hours of administration, there are no remarkable effects of T on protein kinetics. What remains is the elucidation and magnitude of T effects on protein kinetics between the time of administration and the 5 day period that has been reported [1–3].

## Data Availability

The datasets used and analyzed during the current study available from the corresponding author on reasonable request.

## Abbreviations

A: Femoral artery
AA: Amino acid
Ala: Alanine
AUC: Area under the curve
DNS: de novo synthesis
FBR: Fractional breakdown rate
Fin: Amino acid inflow into leg from systemic circulation via femoral artery
Fm,a: Inward amino acid transport from femoral artery info free muscle
Fm,o: Intrecellular amino acid appearance from endogenous sources
Fo,m: Intracellular amino acid utilization
Fout: Amino acid outflow from leg via femoral vein
FSR: Fractional synthesis rate
Fv,a: Direct amino acid ouflow from artery to vein without entering intracellular fluid
Fv,m: Outward amino acid transport from intracellular pool into femoral vein
GC-MS: Gas chromatography-mass spectrometry
IL: Intralipid
IR: Infusion rate
Leu: Leucine
Lys: Lysine
M: Muscle
NB: Net balance
PB: Protein breakdown
PD: Primed
Phe: Phenylalanine
PS: Protein synthesis
SD: Standard deviation
SEM: Standard error of measurement
T: Testosterone
UTMB: University of Texas Medical Branch at Galveston
V: Femoral vein
